# Supplementary material for: Sperm hyperactivation drives a circling-and-wandering swimming behavior
Source: Nat Commun. 2026 Mar 24;17:4475. doi: 10.1038/s41467-026-70143-6 (PMC13187331; doi:10.1038/s41467-026-70143-6)
Supplement: Supplementary file 1 — Supplementary Information text [file 41467_2026_70143_MOESM1_ESM.pdf]

Supplementary Information for "Sperm hyperactivation drives a circling-and-wandering swimming behavior"

Meisam Zaferani[1,2,3,*], Yanis Baouche[4], Yamilka Lago-Alvarez[5], Anish Pandya[4], Soon Hon Cheong[5], Sabine Petry[2], Christina Kurzthaler[4,6,7,*], and Howard A. Stone[1,*]

[1]Department of Mechanical and Aerospace Engineering, Princeton University, Princeton, NJ, USA
[2]Department of Molecular Biology, Princeton University, Princeton, NJ, USA
[3]Omenn-Darling Bioengineering Institute, Princeton University, Princeton, NJ, USA
[4]Max Planck Institute for the Physics of Complex Systems, 01187 Dresden, Germany
[5]Department of Clinical Sciences, College of Veterinary Medicine, Cornell University, Ithaca, NY, USA
[6]Center for Systems Biology Dresden, 01307 Dresden, Germany
[7]Cluster of Excellence Physics of Life, TU Dresden, 01062 Dresden, Germany
[*]Corresponding authors: mzaferani@princeton.edu, ckurzthaler@pks.mpg.de, hastone@princeton.edu

1 Supplementary Tables

	Sample 1			Sample 2			Sample 3			
Chamber ID	1	2	3	1	2	3	1	2	3	4
Number of cells	57	38	49	31	24	38	50	41	59	34
Number of cells with rolling suppressed	53	34	43	29	20	34	48	34	55	33
Number of cells with resumed rolling	14	10	13	7	4	10	11	11	16	10

Table S1. Statistics of suppressed and resumed rolling

Chamber ID	1	2	3	4	5	6	7	8	Total
Number of cells in the chamber	1	2	1	1	2	1	1	1	10
Number of cells with C&W	0	1	0	1	0	1	0	0	3

Table S2. Statistics of circling and wandering in sample 1.

Chamber ID	1	2	3	4	5	6	Total
Number of cells in the chamber	2	1	2	1	1	1	8
Number of cells with C&W	0	1	1	1	0	0	3

Table S3. Statistics of circling and wandering in sample 2.

Chamber ID	1	2	3	4	5	6	7	Total
Number of cells in the chamber	1	2	2	1	2	1	1	10
Number of cells with C&W	1	0	2	1	0	1	1	6

Table S4. Statistics of circling and wandering in sample 3.

Chamber ID	1	2	3	4	5	6	Total
Number of cells in the chamber	1	2	2	2	1	2	10
Number of cells with C&W	0	1	1	0	0	0	2

Table S5. Statistics of circling and wandering in sample 4.

Chamber ID	1	2	3	4	5	6	7	8	Total
Number of cells in the chamber	1	1	2	1	2	1	1	1	10
Number of cells with C&W	1	0	0	1	1	1	0	0	4

Table S6. Statistics of circling and wandering in sample 5.

2 Supplementary figures

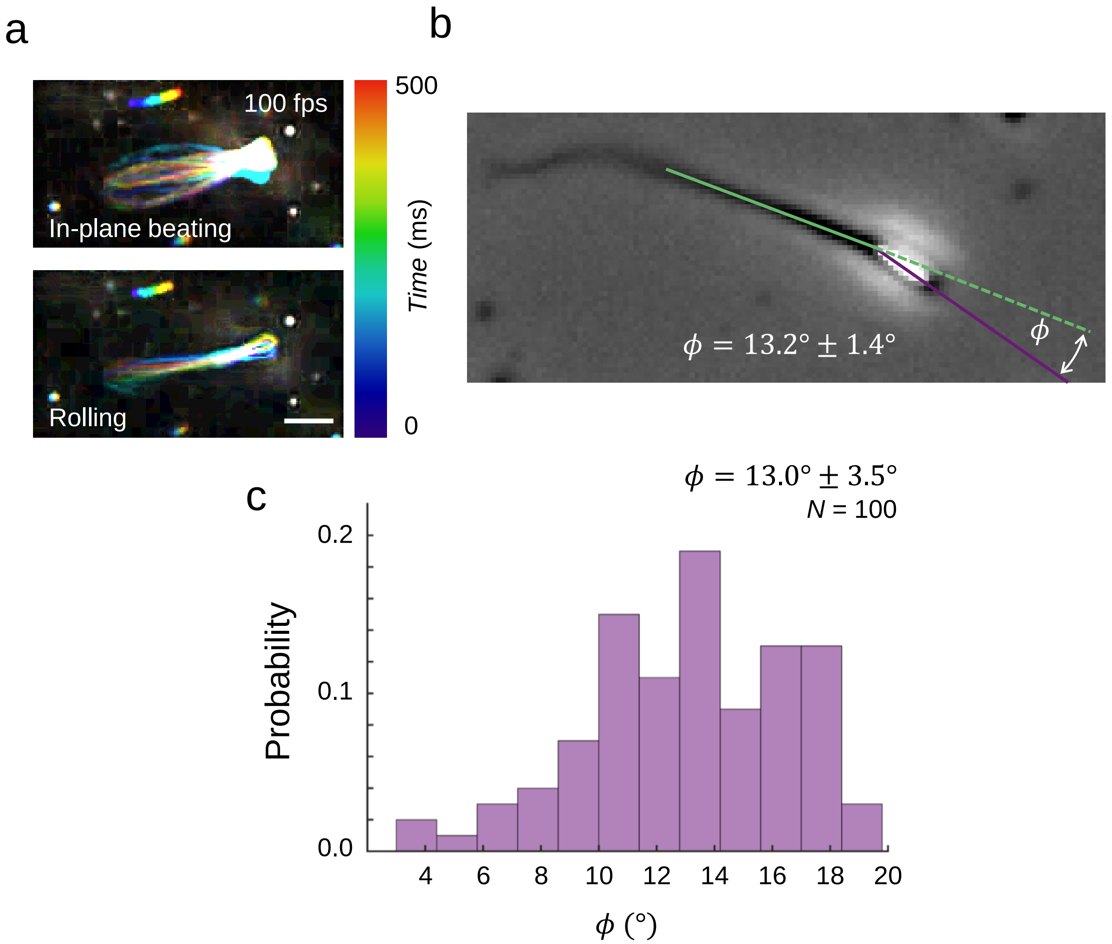

Figure S 1. Sperm head morphology and rolling. (a) Bull sperm motility observed at 100 fps under phase-contrast microscopy. (b) Bull sperm head tilted out of the flagellar beating plane. (c) Histogram of the head angle with respect to the beating plane for $N = 100$ sperm.

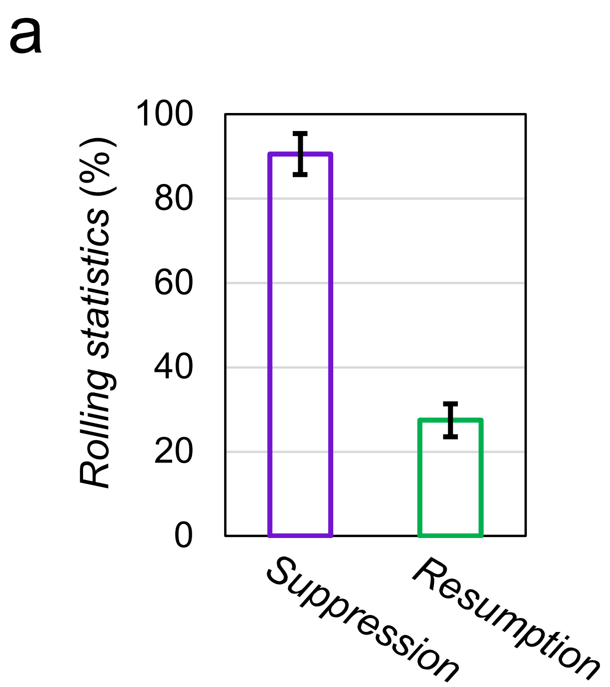

Figure S 2. **Statistics of circling-and-wandering motility.** a) Statistics of sperm rolling suppression and resumption in 1% PAM. b) Average duration of circling and wandering periods.

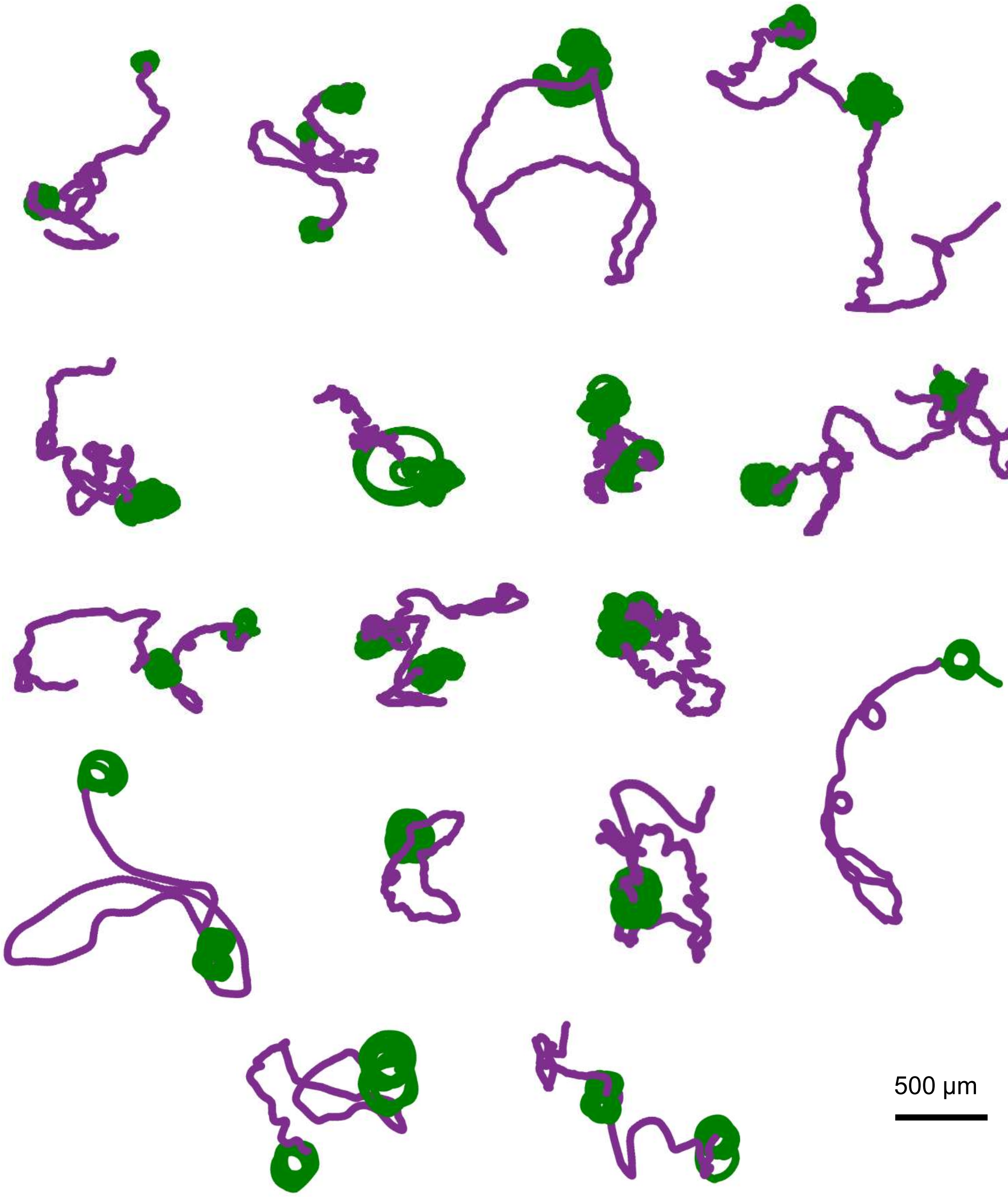

Figure S 3. **Trajectories of sperm circling and wandering.**

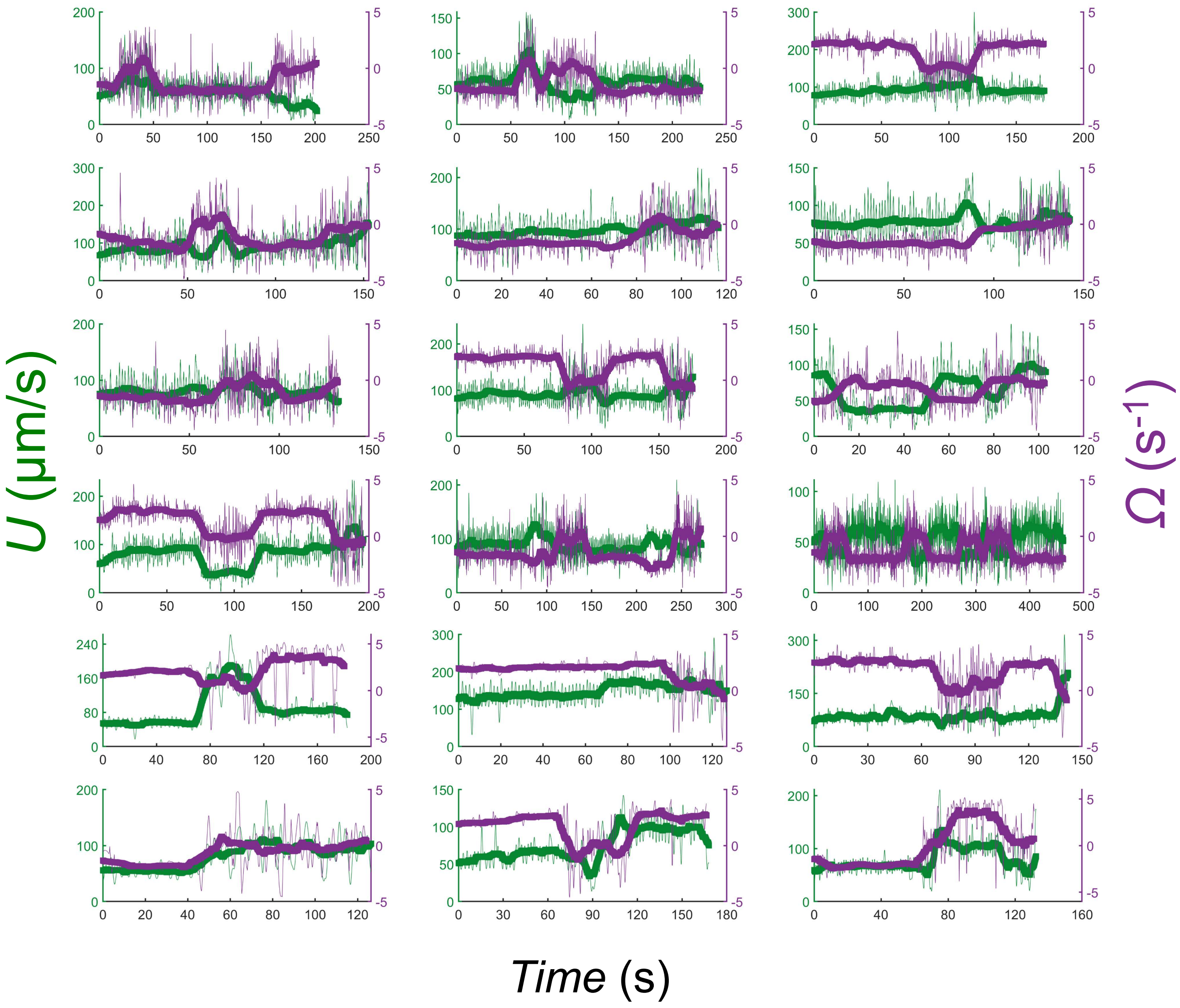

Figure S 4. **Translational and rotational speeds.** Corresponding measurements of translational and rotational speeds for the trajectories shown in Figure S2. The transition between circling and wandering appears in the speed changes and in the suppression of rolling. Real time measurements are shown as thin lines, while the smoothed values are shown as thick lines.

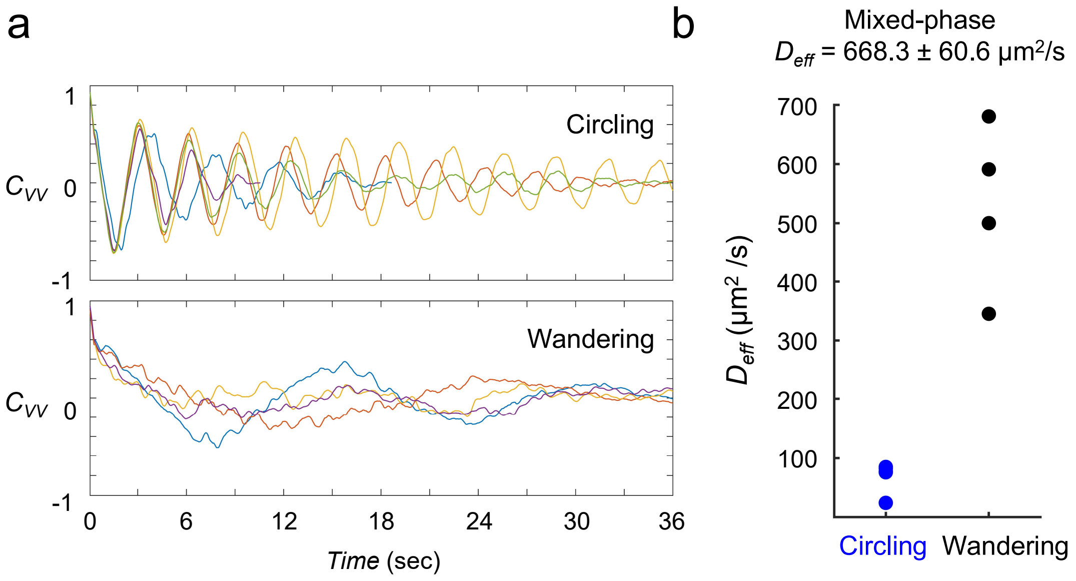

Figure S 5. Constituent circling and wandering periods in mixed motility. (a) Velocity correlation function of constituent phases. Each color represents the trajectory of a constituent phase. (b) Effective diffusivities for each phase and the overall mixed phase.

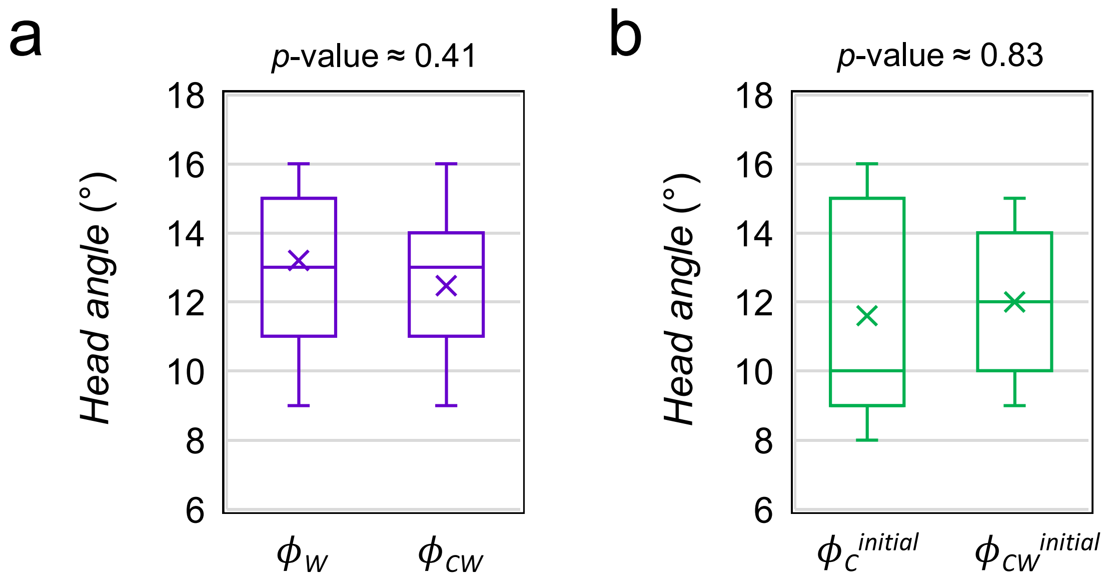

Figure S 6. **Statistics of sperm head angle.** a) Head angle during pure wandering versus during the wandering periods of circling-and-wandering trajectories. b) Initial head angle of cells that later exhibit pure chiral motion versus circling-and-wandering motility in the chamber.

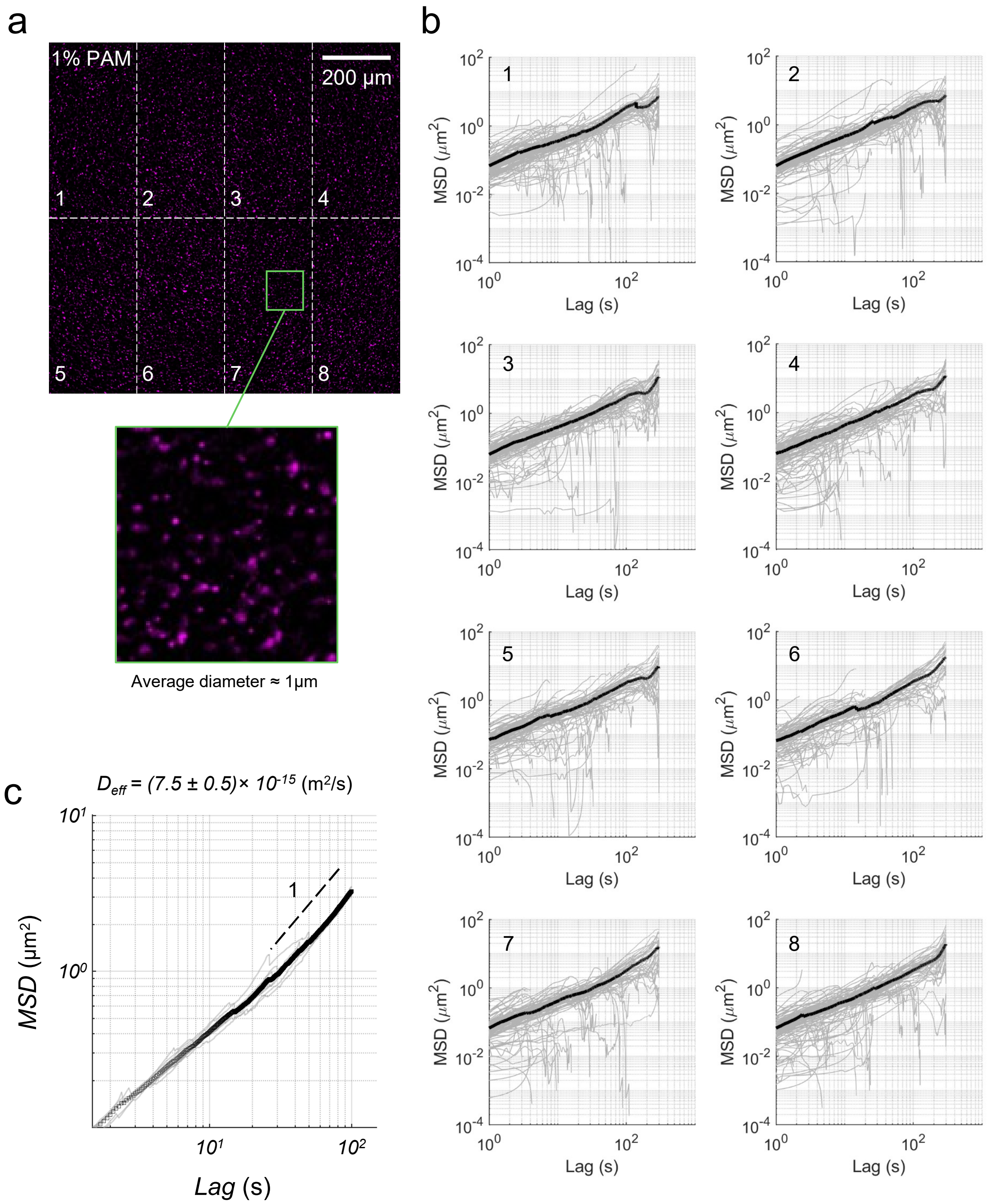

Figure S 7. Microrheological measurement in TALP + 1% PAM. a) Microspheres with an average diameter of 1 μm were added to the solution, and their motion was recorded in eight regions of the chamber. b) MSDs corresponding to tracks acquired at 10 FPS over five minute intervals from more than 100 microspheres in each region. All regions show diffusive behavior at long times. c) Average MSDs for each region collapse to a single master curve.

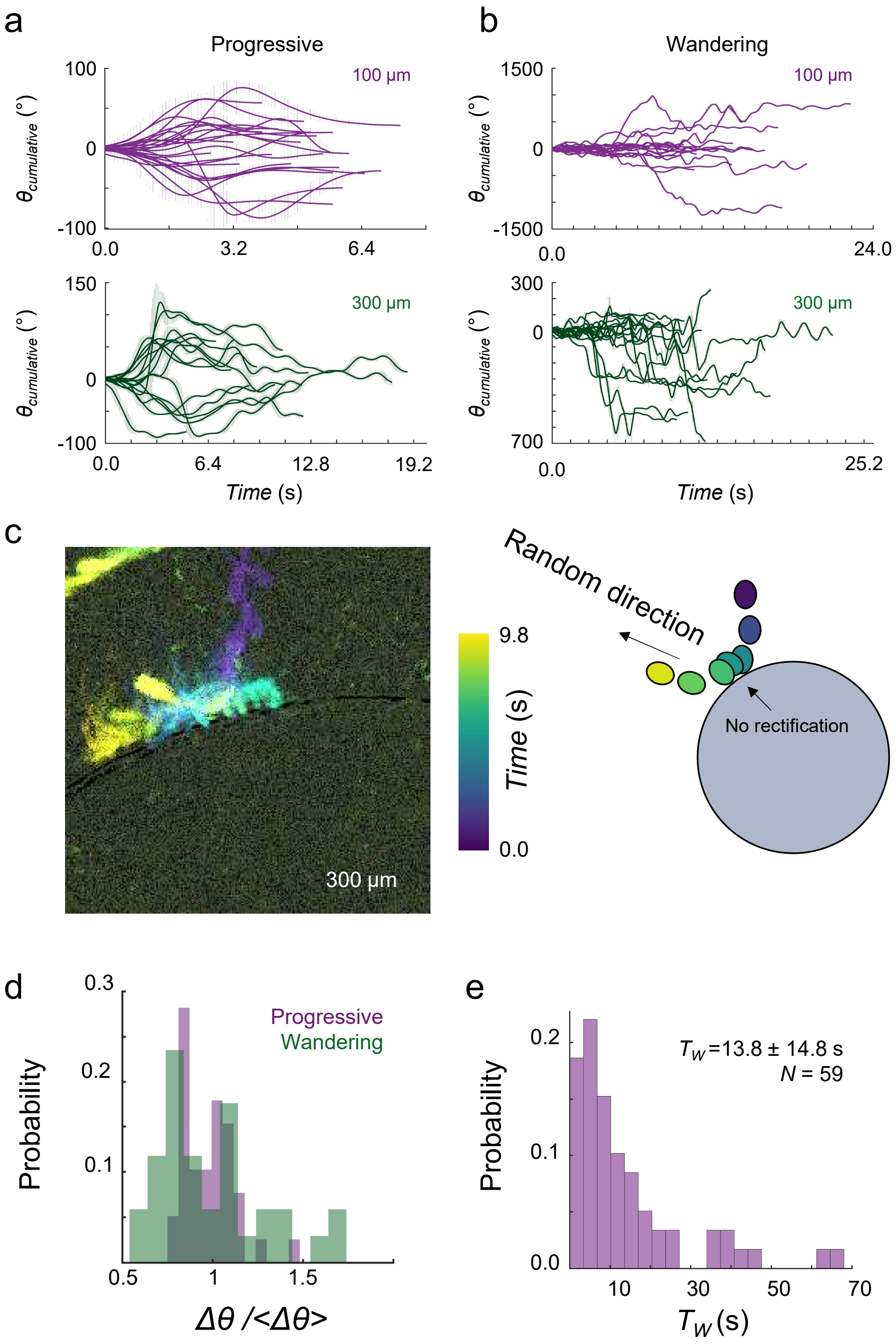

Figure S 8. Wall interactions in the wandering phase. (a) Cumulative deflection angle ($\theta_{\mathrm{cumulative}}$) for (a) progressive and (b) wandering sperm scattered from pillars of diameters $D = 100\,\mu m$ and $D = 300\,\mu m$. (c) In the wandering phase, sperm contact length on the pillar is deterministically set by the deflection angle, while scattering occurs randomly without rectification by the pillar. (d) Histogram of sperm relative deflection angles in progressive and wandering phases. (e) Histogram of T_{W} for $N = 59$ trajectories.

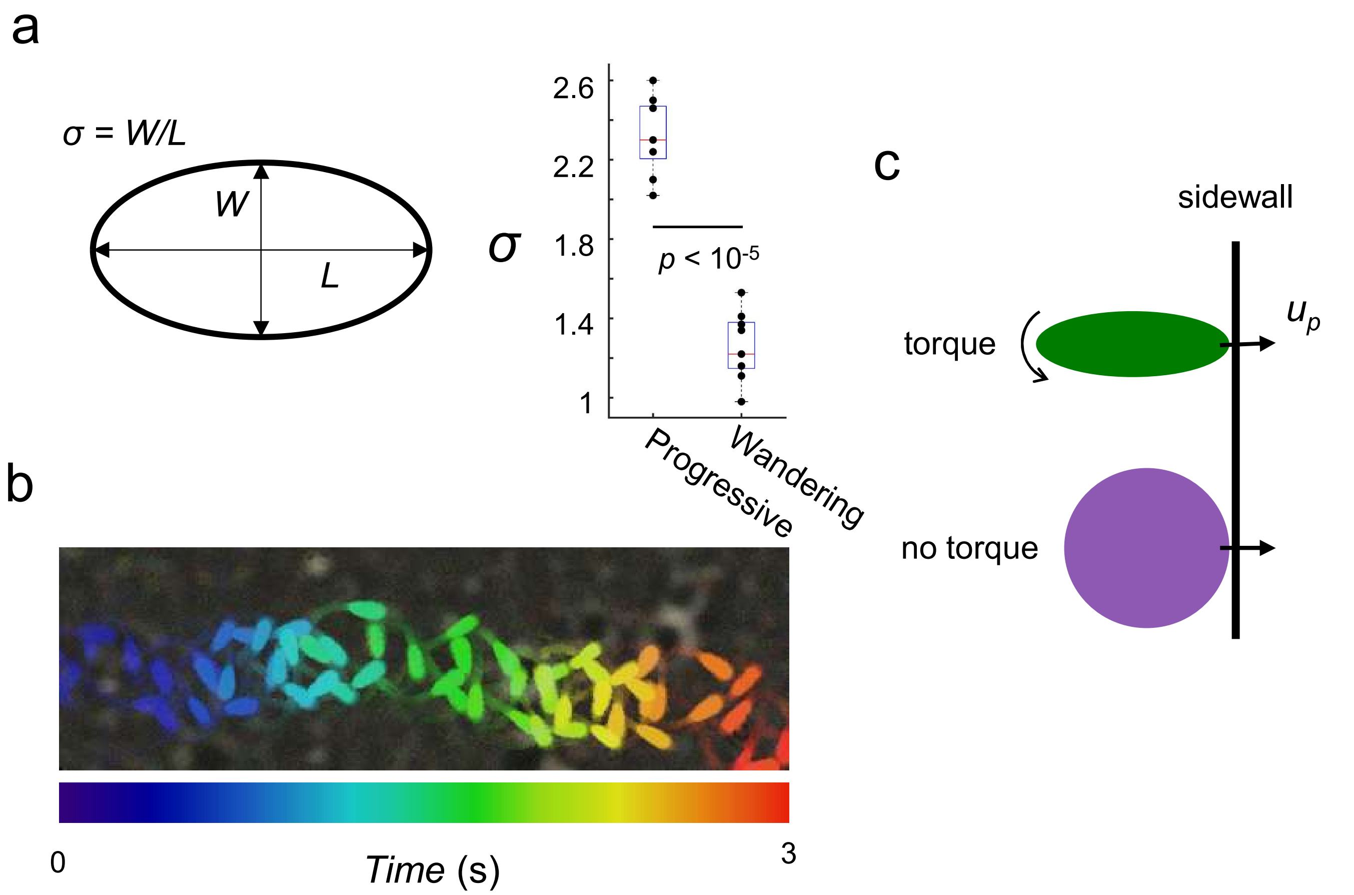

Figure S 9. Sperm aspect ratio. (a) Aspect ratio measured from overlaid frames of sperm over a 600-millisecond period with 60-millisecond intervals, capturing both progressive and wandering phases. (b) Wandering sperm exhibit an effectively spherical shape compared to the elongated oval shape of progressive sperm. (c) High-aspect-ratio, oval-shaped swimmers experience an aligning torque upon collision with a physical boundary, whereas low-aspect-ratio, spherical swimmers experience significantly lower aligning torque.

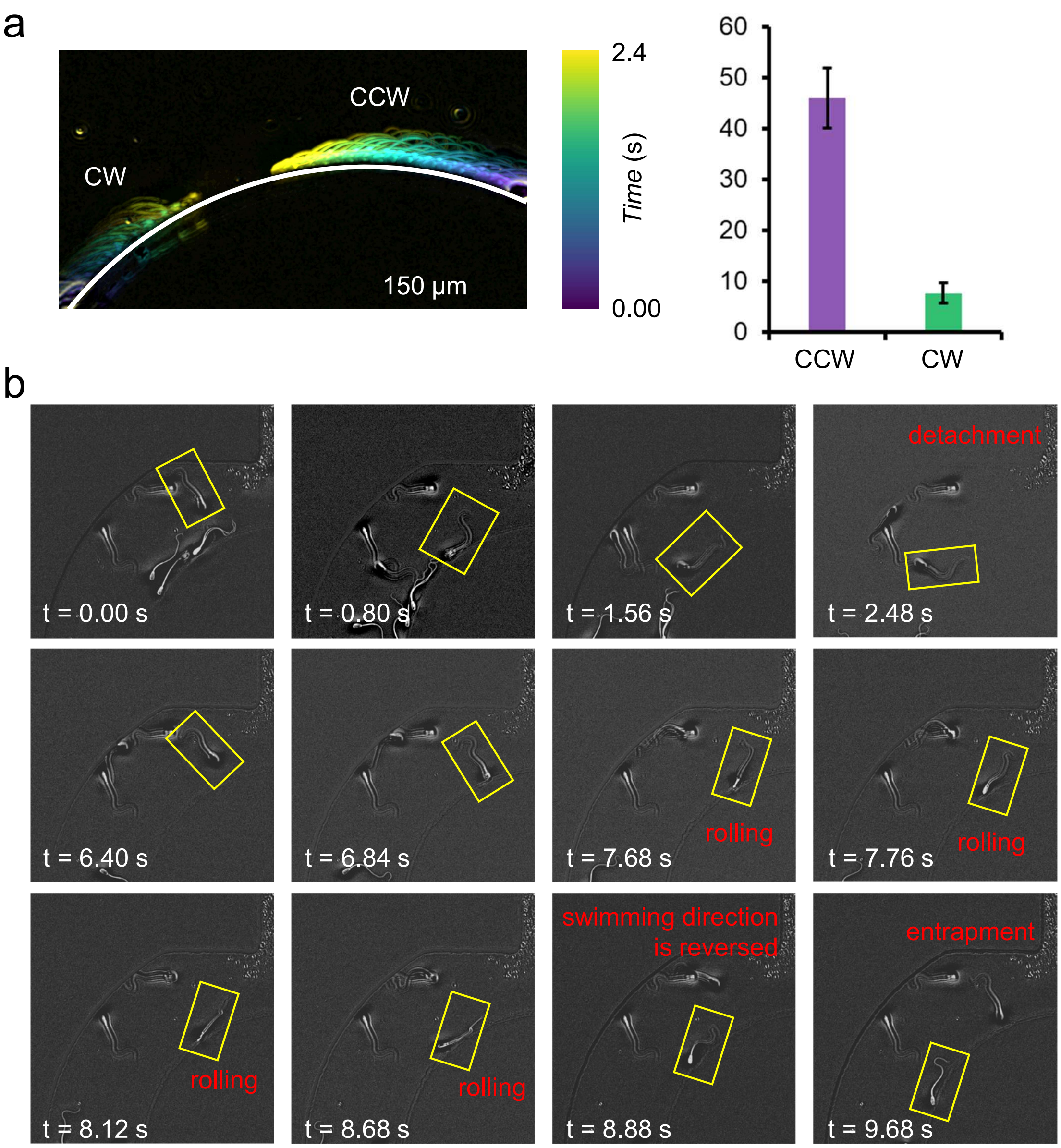

Figure S 10. Characterization of Sperm Entrapment I. (a) Circling sperm with both clockwise (CW) and counterclockwise (CCW) chirality trapped around the $D = 300\,\mu m$ pillar. (b) Transitory rolling events reversed the chirality of sperm circling motion, leading to entrapment around the $D = 300\,\mu m$ pillar.

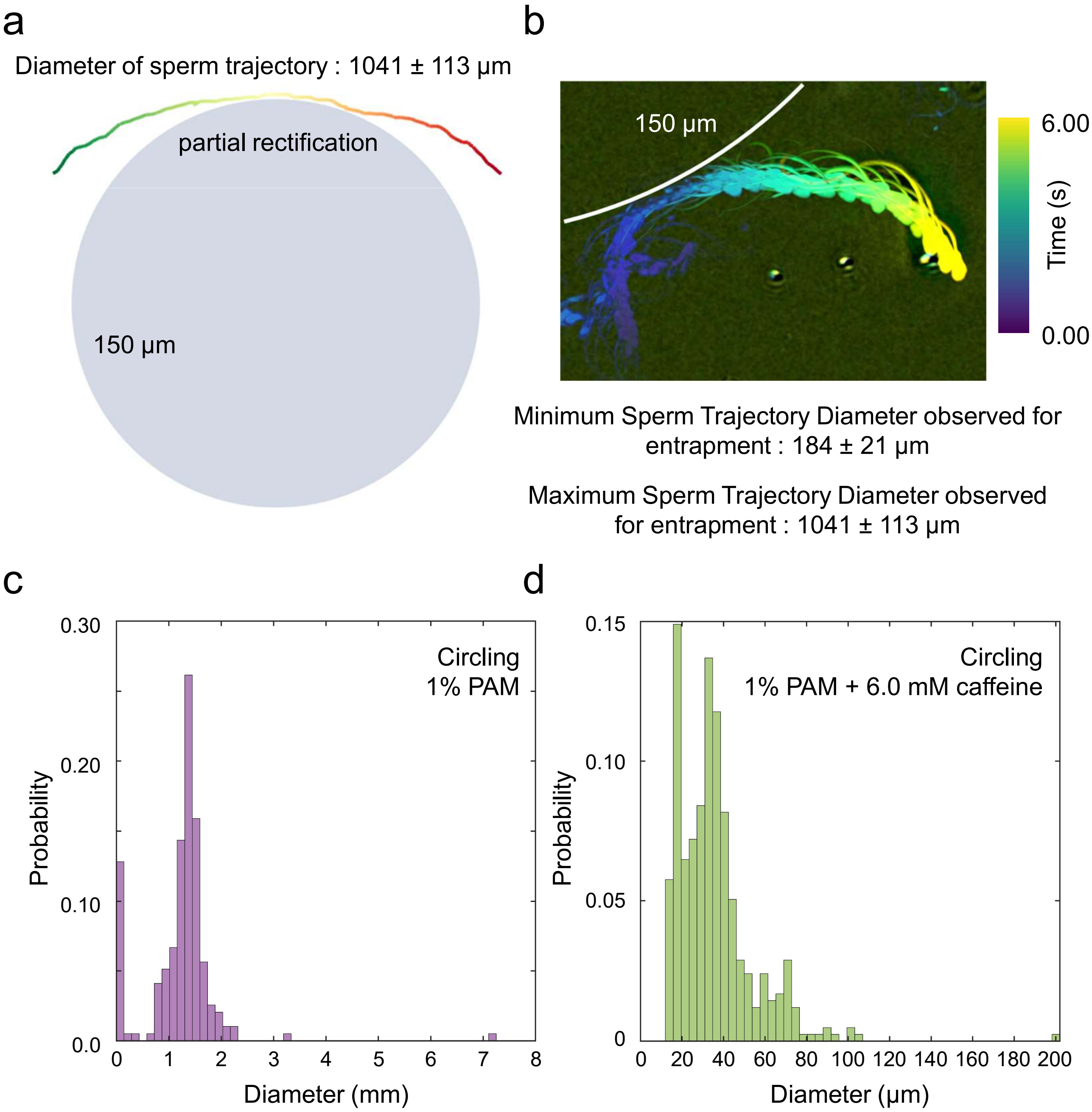

Figure S 11. **Characterization of Sperm Entrapment II.** (a) Trajectory of a non-caffeine-treated circling sperm that is partially rectified by the pillar. (b) Trajectory of a non-caffeine-treated circling sperm that is not rectified by the pillar. Based on these measurements, we estimate the minimum and maximum trajectory diameters required for entrapment around the pillar. (c) Histogram of the sperm trajectory diameter in the circling phase without caffeine treatment. (d) Histogram of the sperm trajectory diameter in the circling phase with caffeine treatment (i.e., hyperactivation).

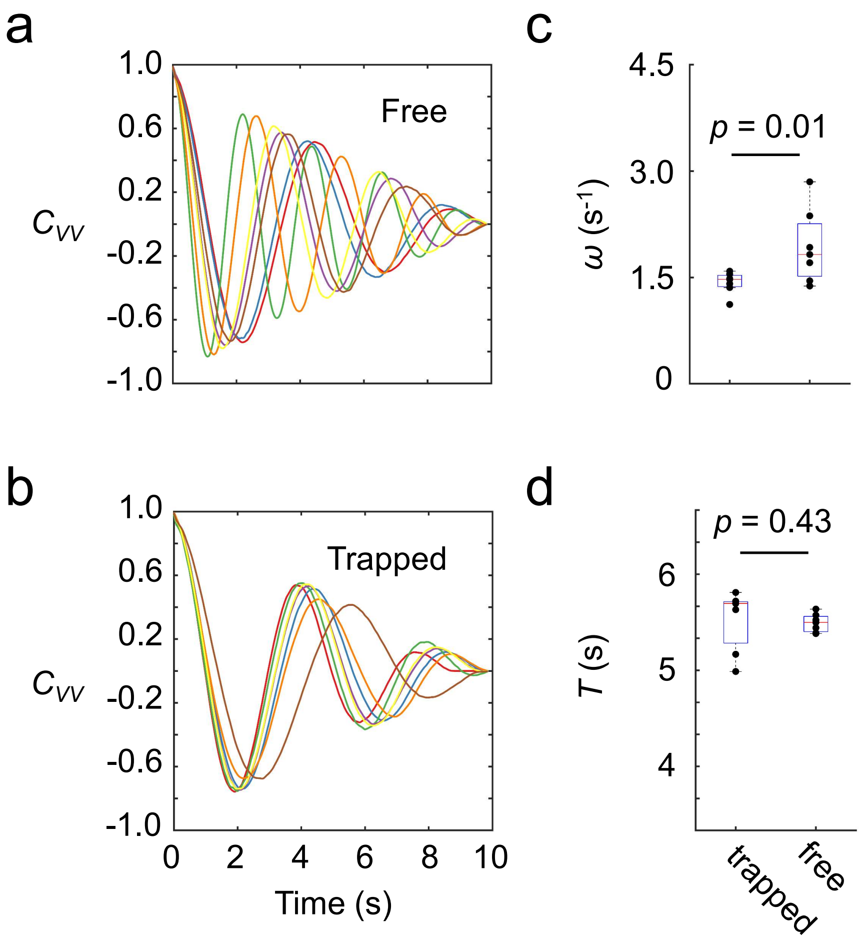

Figure S 12. **Characterization of Sperm Entrapment Around Small Pillars.** (a) Velocity correlation function for individual caffeine-treated circling sperm before entrapment around the pillar. Each color represents an individual cell. (b) Velocity correlation function after entrapment. (c) Corresponding rotational speeds. (d) Corresponding relaxation times.

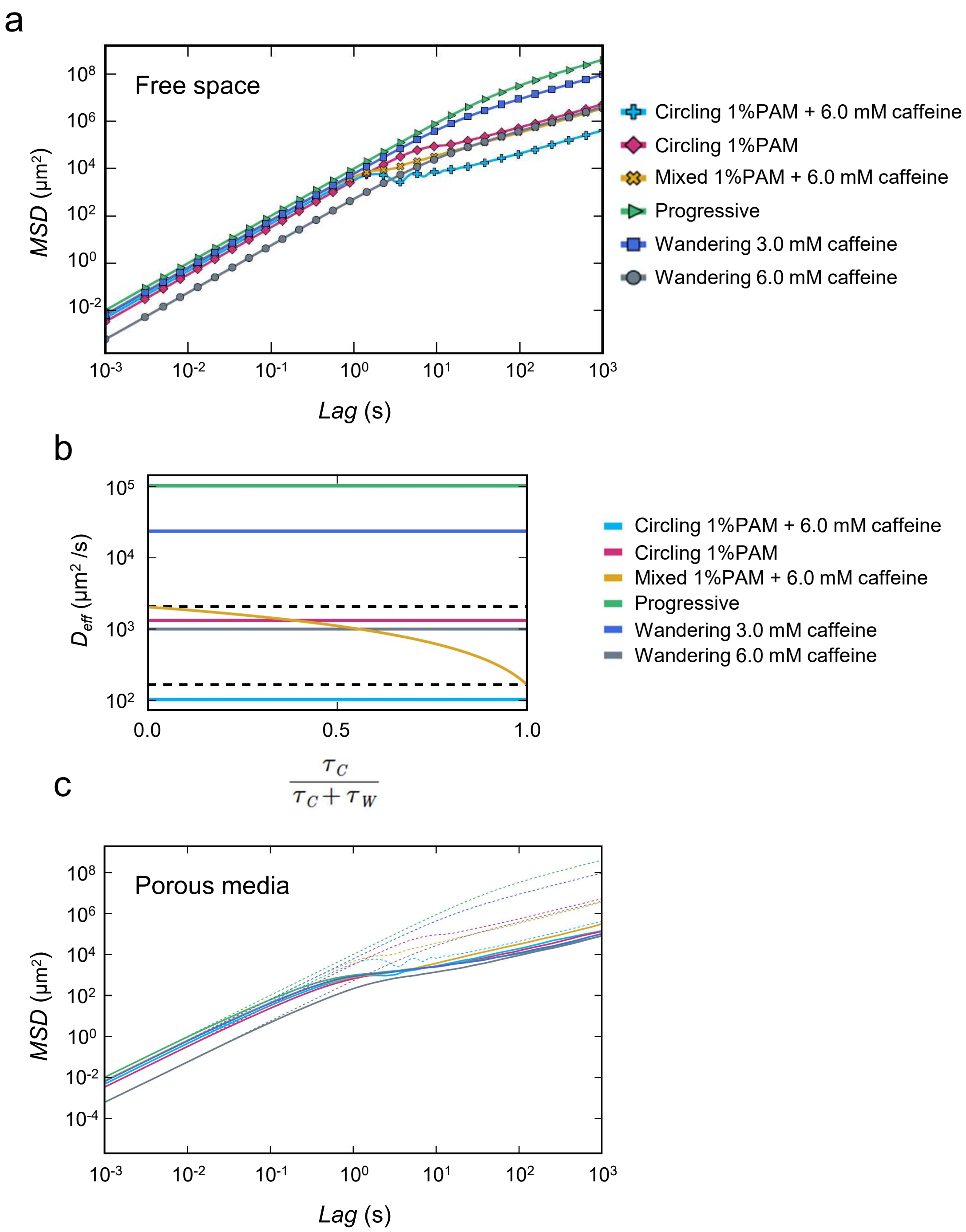

Figure S 13. **Mean-square displacements and effective diffusivity obtained from theory and simulations.** (a) MSDs in free space computed for each motility phase. Lines correspond to the theoretical predictions and symbols to simulations. (b) Effective diffusivity D_{eff} of the mixed phase as a function of the fraction of time spent in the circling phase. (c) MSDs in porous media (solid lines) for $\eta = 0.55$ and mean chord length $l_h/l_c = 0.88$, obtained from simulations. Dashed lines show the theoretical predictions for the MSD in free space for comparison.

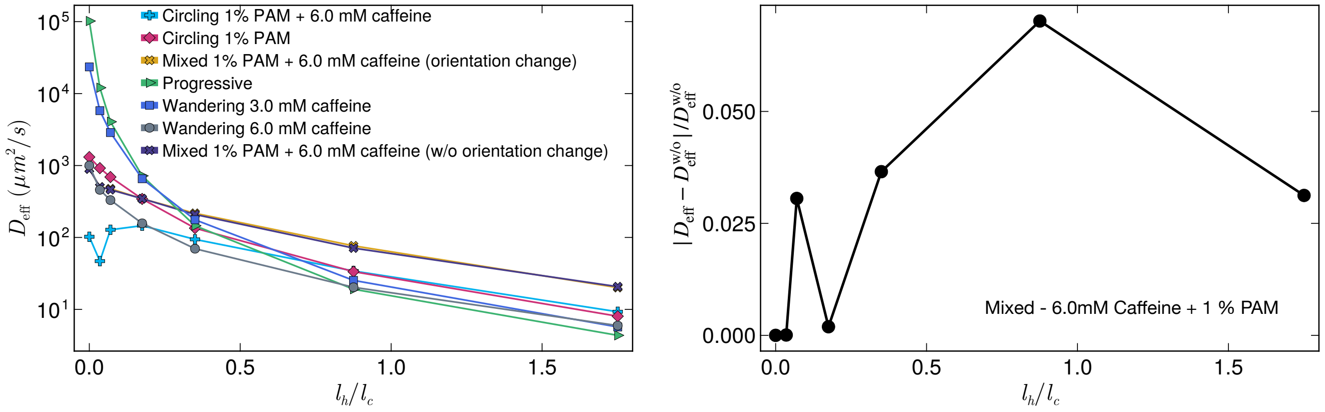

Figure S 14. **Long-time effective diffusivities** $D_{\rm eff.}$ *Left panel*: The yellow symbols correspond to the results for the mixed phase, where the agent does not change its swimming direction between two phases, and the dark purple symbols show the results for agents that change swimming direction randomly at each phase change. *Right panel*: Relative difference between the two models for the mixed phase. $D_{\rm eff}$ corresponds to a random change of orientation at the transition between phases and $D_{\rm eff}^{\rm w/o}$ corresponds to no change in orientation.
