# Supplementary material for: Sperm hyperactivation drives a circling-and-wandering swimming behavior
Source: Nat Commun. 2026 Mar 24;17:4475. doi: 10.1038/s41467-026-70143-6 (PMC13187331; doi:10.1038/s41467-026-70143-6)
Supplement: Supplementary file 2 — Description of Additional Supplementary Files [file 41467_2026_70143_MOESM2_ESM.pdf]

Description of Additional Supplementary Files

Supplementary Movies S1–S7

Legends:

Movie S1: Bull sperm progressive motility in TALP under phase-contrast microscopy, recorded at 100 frames per second. Rolling of sperm around the longitudinal axis is observed by probing the light intensity of the sperm head. The thin edge of the paddle-shaped head appears bright.

Movie S2: Sperm wandering motility in TALP + 6.0 mM caffeine (left) and circling motility in TALP + 1% PAM + 6.0 mM caffeine (right). The chamber radius is 600 μm.

Movie S3: Two examples of sperm circling-and-wandering motility in TALP + 1% PAM + 6.0 mM caffeine. The chamber radius is 600 μm. Top video: recorded at 5 frames per second. Bottom video: recorded at 15 frames per second.

Movie S4: A wandering sperm in TALP + 6.0 mM caffeine detaches from the chamber sidewall. The chamber radius is 600 μm. The video is recorded at 15 frames per second.

Movie S5: Wandering sperm in TALP + 6.0 mM caffeine scatter off pillars with different radii.

Movie S6: Circling sperm in TALP + 1% PAM + 6.0 mM caffeine, trapped around pillars with different radii.

Movie S7: A circling sperm in TALP + 1% PAM changes its chirality through temporary rolling events and becomes trapped around a pillar.
